# Structure of tetra­kis­(μ-deca­noato-κ^2^ *O*:*O*′)bis­[(4-methyl­pyridine-κ*N*)copper(II)], a dimeric copper(II) complex

**DOI:** 10.1107/S2056989020014103

**Published:** 2020-10-30

**Authors:** Monsumi Gogoi, Birinchi Kumar Das

**Affiliations:** a Department of Chemistry, Gauhati University, Guwahati, Assam, India; b Bhattadev University, Bajali, Pathsala, Assam, India

**Keywords:** dimeric, paddle-wheel structure, copper(II), 4-methyl­pyridine, deca­noate, crystal structure

## Abstract

The 4-methyl­pyridine (4-Mepy) based dimeric copper(II) carboxyl­ate complex [Cu_2_(C_10_H_19_O_2_)_4_(C_6_H_7_N)_2_] or [Cu_2_(*μ*-O_2_CC_9_H_19_)_4_(4-Mepy)_2_] crystallizes with triclinic (*P* ) symmetry. The two Cu^II^ ions exhibit a distorted square-pyramidal environment and are connected into a centrosymmetric paddle-wheel dinuclear cluster [Cu⋯Cu = 2.6472 (8) Å] *via* four bridging carboxyl­ate ligands arranged in the *syn–syn* coordination mode. The apical positions around the paddle-wheel copper centers are occupied by the N atoms of the 4-methyl­pyridine ligands. The structure exhibits disorder of the terminal alkyl carbon atoms in the deca­noate chains.

## Abstract 2

The 4-methyl­pyridine (4-Mepy) based dimeric copper(II) carboxyl­ate complex [Cu_2_(C_10_H_19_O_2_)_4_(C_6_H_7_N)_2_] or [Cu_2_(*μ*-O_2_CC_9_H_19_)_4_(4-Mepy)_2_] crystallizes with triclinic (*P* 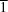) symmetry. The two Cu^II^ ions exhibit a distorted square-pyramidal environment and are connected into a centrosymmetric paddle-wheel dinuclear cluster [Cu⋯Cu = 2.6472 (8) Å] *via* four bridging carboxyl­ate ligands arranged in the *syn–syn* coordination mode. The apical positions around the paddle-wheel copper centers are occupied by the N atoms of the 4-methyl­pyridine ligands. Parts of the deca­noate chains are disordered with occupancy ratios of 0.817 (9):0.183 (9) and 0.65 (5):0.35 (5).

## Chemical context   

Research on metal carboxyl­ates has gained importance in view of their use in the formation of open and porous frameworks and also because of their biological activities and anti­bacterial properties (Smithenry *et al.*, 2003 ▸; Lah *et al.*, 2001 ▸). As the number of carboxyl­ate groups increases, so does the complexity of the coordination behaviour. Carboxyl­ate anions are versatile ligands capable of existing as counter-anions or as ligands coordinating to the metal ions in different modes (Deacon & Philips, 1980 ▸; Tao *et al.*, 2000 ▸; Smithenry *et al.*, 2003 ▸). Copper complexes containing aliphatic/aromatic carb­oxy­lic acid anions as ligands with the general formula [Cu_2_(O_2_C*R*)_4_] have been known to adopt a paddle-wheel structure where four bidentate carboxyl­ato ligands bridge the Cu^II^ centres (Baruah *et al.*, 2015 ▸; Serrano & Sierra, 2000 ▸). Complexes having *R* = a long-chain alkyl group can make the resultant dimeric carboxyl­ates more soluble in organic solvents and hence can be more effective as catalysts in certain reactions (Baruah *et al.*, 2015 ▸). These carboxyl­ates can be prepared either by reaction of basic copper(II) carbonate/acetate with the corresponding carb­oxy­lic acid or by reaction of a copper(II) salt with the sodium salt of the corresponding carb­oxy­lic acid (Hamza & Kickelbick, 2009 ▸; Moncol *et al.*, 2010 ▸; Das & Barman, 2001 ▸). Each Cu^II^ centre has four oxygen atoms forming the basal plane, while the axial position is either occupied by a solvent mol­ecule or by a monodentate nitro­gen base ligand or sometimes by an oxygen atom of another dimeric unit resulting in an oligomeric chain (Agterberg *et al.*, 1998 ▸; Wein *et al.*, 2009 ▸). A few members of the family of dicopper(II) tetra­carboxyl­ates of the type [Cu_2_(μ-O_2_C*R*)_4_ *L*_2_] have been demonstrated to be active homogeneous catalysts in the oxidation of various alcohols. A dinuclear complex, [Cu_2_(*μ*-O_2_CC_5_H_11_)_4_(C_6_N_2_H_4_)_2_] (Baruah *et al.*, 2015 ▸), was reported as having two crystallographically independent Cu^II^ atoms in a distorted square-pyramidal environment.
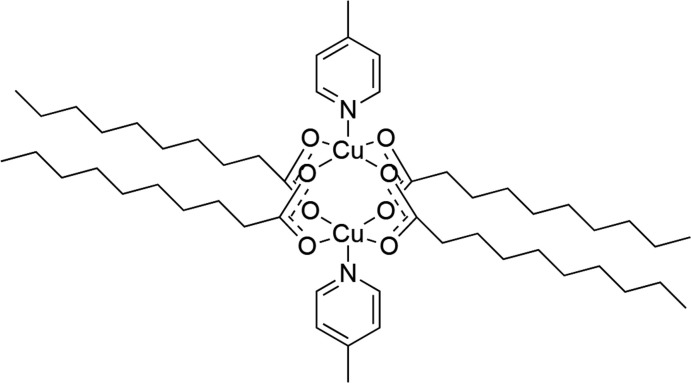


## Structural commentary   

**Figure 1:**
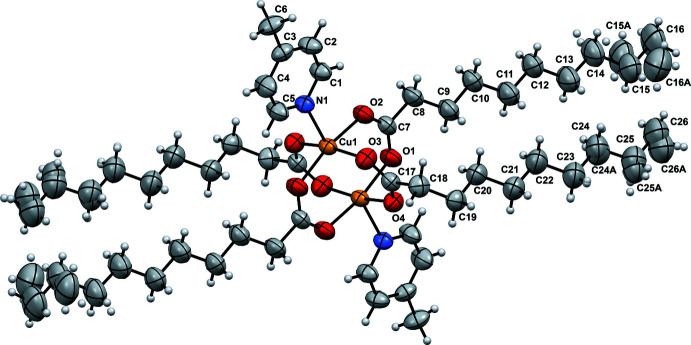
ORTEP diagram of [Cu_2_(μ-O_2_CC_9_H_19_)_4_(4-Mepy)_2_] showing the atom-labelling scheme (ellipsoids drawn at the 50% probability level; unlabelled atoms generated by the symmetry operation 1 − *x*, 1 − *y*, 1 − *z*).

The title compound [Cu_2_(*μ*-O_2_CC_9_H_19_)_4_(4-Mepy)_2_] crystallizes in the triclinic system, space group *P* 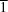. The complex has a centrosymmetric structure and consists of a copper(II) dimer having a paddle-wheel structure. The asymmetric unit comprises a Cu^II^ ion coordinated by the N atom of 4-methyl­pyridine and by two deprotonated O-monodentate deca­noate ligands. The two Cu^II^ ions are bridged by four carboxyl­ate ligands in the *syn–syn* coordination mode, resulting in a distorted square-pyramidal environment with the four O atoms forming the square basal plane and the two pyridyl-N atoms of the 4-Mepy ligands occupying the apical positions. The mol­ecular structure of the complex is shown in Fig. 1 ▸.

The Cu⋯Cu [2.6472 (8)], Cu—O (average) [1.9740 (12)], and Cu—N [2.1680 (14) Å] distances are comparable to those observed for structurally similar Cu^II^ dimers with a [Cu_2_(*μ*-O_2_C*R*)_4_ *L*_2_]-type structure, [Cu_2_(*μ*-O_2_CCMe_3_)_4_(NC_5_H_3_(2-NH_2_)(6-CH_3_))_2_] (Fomina *et al.*, 2010 ▸) and [Cu_2_(μ-O_2_CC_6_H_5_)_4_(py)_2_] (Iqbal *et al.*, 2014 ▸). The Cu⋯Cu distance in the title complex was found to be slightly longer than in the copper(II) carboxyl­ate complex [Cu_2_(*μ*-O_2_CC_5_H_11_)_4_(4-NCpy)_2_] [2.6055 (9) Å; Baruah *et al.*, 2015 ▸) and in [Cu_2_(*μ-*O_2_CC_9_H_19_)_4_(NC_5_H_4_CO_2_C_12_H_25_)_2_] [2.615 (1) Å; Rusjan *et al.*, 2000 ▸). The Cu—-N bond in the title complex is slightly shorter than those reported by Rusjan *et al.* (2000 ▸) and Petric *et al.* (1993 ▸). The difference between the Cu⋯Cu and Cu—N distances and those for related complexes is probably due to the difference in the basicity of the pyridinic group in the apical position of the core. The hydrogen atoms at positions 2 and 6 of the aromatic ring establish intra­molecular C—H⋯O inter­actions with the closely placed carboxyl­ate oxygen atoms (Table 1 ▸).

In the title complex, the two oppositely placed deca­noate alkyl chains adopt a fully elongated zigzag conformation, whereas the other pair is distorted, aligning parallel to the first one after a *gauche* conformation at the C18—C19 bond (Rusjan *et al.*, 2000 ▸). This arrangement probably occurs to facilitate efficient packing. The terminal ends of both pairs of alkyl chains are disordered and were modelled as described in the *Refinement* section.

## Supra­molecular features   

**Figure 2:**
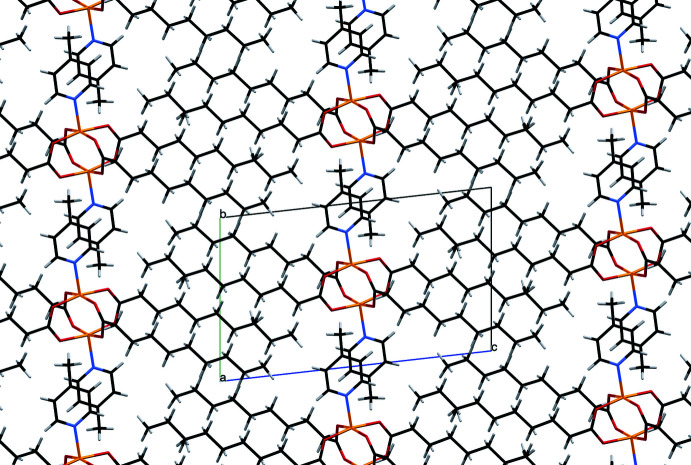
Packing of [Cu_2_(*μ*-O_2_CC_9_H_19_)_4_(4-Mepy)_2_] viewed along the *a* axis (disorder not shown)

**Figure 3:**
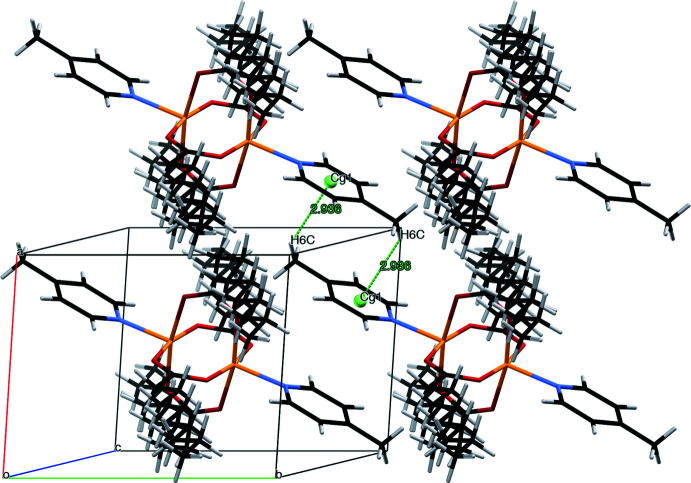
C6—H6*C*⋯π inter­actions in [Cu_2_(μ-O_2_CC_9_H_19_)_4_(4-Mepy)_2_] (disorder not shown)

There is no strong inter­molecular hydrogen bonding in the title complex because of the absence of sufficiently polar hydrogen atoms. The supra­molecular structure of the complex shows two different sets of dimers. One involves a pair of symmetry-related C18—H18⋯O3 inter­actions (Table 1 ▸) that form dimers and give rise to the formation of infinite chains along the *a*-axis direction. The second one involves dimers linked by a pair of C6—H6*B*⋯O2 inter­actions that form infinite chains along the *b*-axis direction. The inter­linking between them gives rise to the crystal packing in the complex, as shown in Fig. 2 ▸. The crystal packing is also supported by C6—H6*C*⋯π inter­actions between a pyridine ring-bound methyl group and the pyridine ring (–*x*, 2 – *y*, 1 – *z*) of a neighbouring 4-Mepy unit with an H⋯centroid distance of 2.94 Å and C—H⋯centroid angle of 134° (Fig. 3 ▸). At the same time, the centroid⋯centroid distances of 4.4183 (14) Å and 4.6957 (15) Å with slippage of 2.909 and 2.913 Å, respectively, between neighbouring pyridine rings (Fig. 3 ▸) are too long for meaningful π–π inter­actions (Tsuzuki *et al.*, 2002 ▸). More details on the mutual arrangement of the pyridine rings can be found in Table 2 ▸.

## Database survey   

A survey of the Cambridge Structural Database (CSD version 2020.2; Groom *et al.*, 2016 ▸) for dimeric copper complexes of alkyl carboxyl­ates revealed that most of the complexes adopt a paddle-wheel structure with a slightly distorted square-pyramidal environment around the Cu^II^ ions. The crystal structure of tetra­kis­(*μ*-hepta­noato-*O,O′*)bis­(nicotinamide)­dicopper(II) (CSD refcodes: CAYHIT and CAYHIT01; Kozlevcar *et al.*, 1999 ▸) and tetra­kis­(*μ*-octa­noato-*O,O′*)bis­(N,N-di­ethyl­nicotinamide)­dicopper(II) (GUH­JIC; Kozlevcar *et al.*, 2000 ▸) were reported as having normal zigzag as well as distorted alkyl chains. Riesco and co-workers reported on the preparation of three polymorphs of Cu^II^ deca­noate, which differ in the cell parameters and the packing of chains following crystallization using different solvents (CUDECN01 and CUDECN02; Riesco *et al.*, 2008 ▸, 2015 ▸). In the do­decyl­nicotinate bis-adduct of a centrosymmetric dinuclear copper deca­noate (XADREZ; Rusjan *et al.*, 2000 ▸) with average Cu—O, Cu—N and Cu⋯Cu distances of 1.960 (6), 2.183 (3) and 2.615 (1) Å, respectively, the alkyl chains in the complex lead to the formation of two different layers along the crystal: one defined by the polar copper carboxyl­ate cores and the second, non-polar one containing the alkyl chains. The Cu^II^ octa­noate adduct with pyridine, *viz.* tetra­kis­(*μ*-octa­noato-O,O′)bis­(pyridine)­dicopper(II) (HEDNIN; Petric *et al.*, 1993 ▸) has a Cu—N bond of 2.194 (4) Å. The dimeric structure of copper(II) hexa­noate with 2-amino­pyridine (QUCQIO; Lah *et al.*, 2001 ▸) is of the typical dinuclear paddle-wheel type and features intra­molecular as well as inter­molecular hydrogen bonds as a result of the presence of the NH_2_ group. Here all the hydro­carbon chains of the octa­noate are found to be distorted and not in the typical zigzag conformation.

## Synthesis and crystallization   

All reagents were purchased from E. Merck and used as received without further purification. CuSO_4_·5H_2_O (0.4994 g, 2.0 mmol) and sodium deca­noate (0.7708 g, 4.0 mmol) were stirred in 25 mL of methanol. After 30 minutes, 4-methyl pyridine (0.1863 g, 2.0 mmol) was added to the reaction mixture, and stirring was continued for 3 h. The resulting green product was filtered off, washed repeatedly with small volumes of methanol and dried in a vacuum desiccator over fused CaCl_2_ (yield 0.8180 g, 82%). The product was dissolved in aceto­nitrile to give a greenish homogeneous solution, which was allowed to concentrate by evaporation at room temperature. Single crystals suitable for X-ray diffraction analysis were obtained from this solution after one day and were collected by filtration. The compound is insoluble in water but soluble in methanol and aceto­nitrile.

IR spectroscopic data (KBr disc, cm^−1^): *ν*_asym_ (COO^−^) 1580, ν_sym_ (COO^−^) 1381, ν_stretch_ (C—H) 2800–2950, ν_stretch_ (py) 1682, 1489, 1445.

## Refinement   

Crystal data, data collection and structure refinement details are summarized in Table 3 ▸.

C-bound hydrogen atoms were placed in idealized positions with C—H = 0.95–0.99 Å, and refined as riding with *U*_iso_(H) = 1.2*U*_eq_(C) or 1.5*U*_eq_(C-meth­yl). The twofold disordered parts of the deca­noate chains (C15–C16, C24–C26 and C15*A*–C16*A*, C24*A*–C26*A*) have been completed through successive electron density difference-Fourier maps and were refined with a sum of their occupancies restrained to unity using geometry (SAME) and *U* ^ij^ restraints (SIMU and RIGU) implemented in *SHELXL*. The refinement converged with the relative occupancies of 0.817 (9) and 0.183 (9) for the C15–C16 section and 0.65 (5) and 0.35 (5) for the C24–C26 section.

## Supplementary Material

Crystal structure: contains datablock(s) I. DOI: 10.1107/S2056989020014103/jq2001sup1.cif

Structure factors: contains datablock(s) I. DOI: 10.1107/S2056989020014103/jq2001Isup2.hkl

CCDC reference: 2039945

Additional supporting information:  crystallographic information; 3D view; checkCIF report

## Figures and Tables

**Table 1:** Hydrogen-bond geometry (Å, °) *Cg* is the centroid of the N1/C1–C5 ring.

*D*—H⋯*A*	*D*—H	H⋯*A*	*D*⋯*A*	*D*—H⋯*A*
C6—H6*B*⋯O2^i^	0.96	2.73	3.577	148
C18—H18*A*⋯O3^ii^	0.97	2.90	3.841	163
C6—H6*C*⋯*Cg*1^iii^	0.96	2.94	3.665 (2)	134

Symmetry codes: (i) 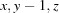; (ii) 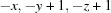; (iii) 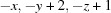.

**Table 2:** Geometry (Å, °) of the stacking of the pyridine rings *Cg*(*I*) = centroid of ring *I*; α = dihedral angle between planes *I* and *J*; β = angle between *Cg*(*I*)⋯*Cg*(*J*) vector and normal to plane *I*; γ = angle between *Cg*(*I*)⋯*Cg*(*J*) vector and normal to plane *J*; *Cg*⋯*Cg* = distance between ring centroids; *Cg*(*I*)_Perp_ = perpendicular distance of *Cg*(*I*) on ring *J*; *Cg*(*J*)_Perp_ = perpendicular distance of *Cg*(*J*) on ring *I*; slippage = distance between *Cg*(*I*) and perpendicular projection of *Cg*(*J*) on ring *I*.

*Cg*(*I*)	*Cg*(*J*)	*Cg*⋯*Cg*	α	β	γ	*Cg*(*I*)_Perp_	*Cg*(*J*)_Perp_	Slippage
*Cg*1	*Cg*1^i^	4.4183 (14)	0	41.2	41.2	3.3258 (8)	3.326	2.909
*Cg*1	*Cg*1^ii^	4.6957 (15)	0	38.3	38.3	−3.6832 (8)	−3.683	2.913

Symmetry codes: (i) 1 − *x*, 2 − *y*, 1 − *z*; (ii) −*x*, 2 − *y*, 1 − *z*.

**Table 3:** Experimental details

Crystal data	
Chemical formula	[Cu_2_(C_10_H_19_O_2_)_4_(C_6_H_7_N)_2_]
*M*_r_	998.33
Crystal system, space group	Triclinic, *P* 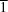
Temperature (K)	293
*a*, *b*, *c* (Å)	8.3146 (17), 10.210 (2), 17.151 (3)
α, β, γ (°)	83.27 (3), 83.78 (3), 86.69 (3)
*V* (Å^3^)	1435.9 (5)
*Z*	1
Radiation type	Mo *K*α
μ (mm^−1^)	0.79
Crystal size (mm)	0.38 × 0.32 × 0.24

Data collection	
Diffractometer	Bruker SMART APEXII
Absorption correction	Multi-scan (*SADABS*; Sheldrick, 2016 ▸)
*T*_min_, *T*_max_	0.752, 0.825
No. of measured, independent and observed [*I* > 2σ(*I*)] reflections	28267, 6789, 5914
*R*_int_	0.029
(sin θ/λ)_max_ (Å^−1^)	0.658

Refinement	
*R*[*F* ^2^ > 2σ(*F* ^2^)], *wR*(*F* ^2^), *S*	0.031, 0.093, 1.05
No. of reflections	6789
No. of parameters	341
No. of restraints	136
H-atom treatment	H-atom parameters constrained
Δρ_max_, Δρ_min_ (e Å^−3^)	0.24, −0.28

Computer programs: *APEX2* and *SAINT* (Bruker, 2004 ▸), *SHELXT2014/5* (Sheldrick, 2015*a* ▸), *SHELXL2017/1* (Sheldrick, 2015*b* ▸), *ORTEP*III (Burnett *et al.*, 1996 ▸), *ORTEP-3 for Windows* (Farrugia, 2012 ▸), *Mercury* (Macrae *et al.*, 2020 ▸) and *publCIF*.

## supplementary crystallographic information

### Crystal data

**Table:**

[Cu_2_(C_10_H_19_O_2_)_4_(C_6_H_7_N)_2_]	*Z* = 1
*M**_r_* = 998.33	*F*(000) = 538
Triclinic, *P*1	*D*_x_ = 1.155 Mg m^−^^3^
*a* = 8.3146 (17) Å	Mo *K*α radiation, λ = 0.71073 Å
*b* = 10.210 (2) Å	Cell parameters from 11736 reflections
*c* = 17.151 (3) Å	θ = 3.4–27.9°
α = 83.27 (3)°	µ = 0.79 mm^−^^1^
β = 83.78 (3)°	*T* = 293 K
γ = 86.69 (3)°	Prism, green
*V* = 1435.9 (5) Å^3^	0.38 × 0.32 × 0.24 mm

### Data collection

**Table:**

Bruker SMART APEXII diffractometer	6789 independent reflections
Radiation source: X-ray tube	5914 reflections with *I* > 2σ(*I*)
Detector resolution: 8.333 pixels mm^-1^	*R*_int_ = 0.029
phi and ω scans	θ_max_ = 27.9°, θ_min_ = 2.2°
Absorption correction: multi-scan (SADABS; Sheldrick, 2016)	*h* = −10→10
*T*_min_ = 0.752, *T*_max_ = 0.825	*k* = −13→13
28267 measured reflections	*l* = −22→22

### Refinement

**Table:**

Refinement on *F*^2^	Primary atom site location: structure-invariant direct methods
Least-squares matrix: full	Secondary atom site location: difference Fourier map
*R*[*F*^2^ > 2σ(*F*^2^)] = 0.031	Hydrogen site location: inferred from neighbouring sites
*wR*(*F*^2^) = 0.093	H-atom parameters constrained
*S* = 1.05	*w* = 1/[σ^2^(*F*_o_^2^) + (0.0528*P*)^2^ + 0.1938*P*] where *P* = (*F*_o_^2^ + 2*F*_c_^2^)/3
6789 reflections	(Δ/σ)_max_ = 0.001
341 parameters	Δρ_max_ = 0.24 e Å^−^^3^
136 restraints	Δρ_min_ = −0.28 e Å^−^^3^

### Special details

**Table:**

Geometry. All esds (except the esd in the dihedral angle between two l.s. planes) are estimated using the full covariance matrix. The cell esds are taken into account individually in the estimation of esds in distances, angles and torsion angles; correlations between esds in cell parameters are only used when they are defined by crystal symmetry. An approximate (isotropic) treatment of cell esds is used for estimating esds involving l.s. planes.

### Fractional atomic coordinates and isotropic or equivalent isotropic displacement parameters (Å^2^)

**Table:**

	*x*	*y*	*z*	*U*_iso_*/*U*_eq_	Occ. (<1)
Cu1	0.45017 (2)	0.62493 (2)	0.48461 (2)	0.04209 (7)	
O1	0.39168 (16)	0.56895 (12)	0.38513 (7)	0.0601 (3)	
C1	0.3374 (2)	0.90403 (17)	0.52306 (11)	0.0590 (4)	
H1	0.382259	0.874290	0.569653	0.071*	
N1	0.35480 (15)	0.82692 (13)	0.46522 (8)	0.0475 (3)	
O2	0.47530 (16)	0.35858 (12)	0.41178 (7)	0.0582 (3)	
C2	0.2564 (2)	1.02557 (17)	0.51769 (12)	0.0638 (4)	
H2	0.248104	1.075746	0.559955	0.077*	
O3	0.24360 (13)	0.56207 (12)	0.54010 (8)	0.0591 (3)	
C3	0.1879 (2)	1.07281 (15)	0.45035 (11)	0.0556 (4)	
C4	0.2067 (3)	0.9932 (2)	0.39042 (12)	0.0752 (6)	
H4	0.163142	1.020798	0.343200	0.090*	
O4	0.32791 (13)	0.35240 (11)	0.56677 (7)	0.0552 (3)	
C5	0.2899 (3)	0.87244 (19)	0.40001 (11)	0.0694 (5)	
H5	0.300801	0.820657	0.358442	0.083*	
C7	0.41767 (19)	0.45288 (16)	0.36848 (9)	0.0500 (3)	
C6	0.0953 (2)	1.20324 (17)	0.44357 (14)	0.0724 (5)	
H6A	0.045278	1.214739	0.395187	0.109*	
H6B	0.167935	1.272921	0.443779	0.109*	
H6C	0.013311	1.205630	0.487395	0.109*	
C9	0.3653 (3)	0.5333 (2)	0.22662 (11)	0.0711 (5)	
H9A	0.472448	0.568107	0.215510	0.085*	
H9AB	0.292583	0.602767	0.245971	0.085*	
C8	0.3706 (3)	0.4222 (2)	0.28978 (11)	0.0717 (5)	
H8A	0.446459	0.354453	0.270772	0.086*	
H8AB	0.264490	0.385065	0.298801	0.086*	
C10	0.3113 (3)	0.4998 (2)	0.15057 (11)	0.0728 (5)	
H10A	0.385633	0.431797	0.130704	0.087*	
H10B	0.205417	0.462754	0.162061	0.087*	
C11	0.3015 (4)	0.6112 (3)	0.08706 (13)	0.0888 (7)	
H11A	0.407685	0.647779	0.075678	0.107*	
H11B	0.228143	0.679476	0.107449	0.107*	
C12	0.2472 (3)	0.5818 (2)	0.01114 (12)	0.0835 (6)	
H12A	0.319653	0.513073	−0.009227	0.100*	
H12B	0.140181	0.546604	0.022153	0.100*	
C13	0.2403 (4)	0.6942 (3)	−0.05142 (14)	0.1024 (8)	
H13A	0.168702	0.762937	−0.030466	0.123*	
H13B	0.347571	0.728921	−0.062123	0.123*	
C14	0.1865 (4)	0.6696 (3)	−0.12709 (14)	0.1044 (9)	
H14A	0.076444	0.640436	−0.117652	0.125*	0.817 (9)
H14B	0.254378	0.598404	−0.147625	0.125*	0.817 (9)
H14C	0.118506	0.594535	−0.113812	0.125*	0.183 (9)
H14D	0.283612	0.636969	−0.156718	0.125*	0.183 (9)
C15	0.1916 (8)	0.7884 (5)	−0.1892 (3)	0.1149 (17)	0.817 (9)
H15A	0.127014	0.860907	−0.168209	0.138*	0.817 (9)
H15B	0.302398	0.815556	−0.200520	0.138*	0.817 (9)
C16	0.1308 (8)	0.7626 (7)	−0.2638 (2)	0.146 (2)	0.817 (9)
H16A	0.196912	0.693389	−0.286207	0.218*	0.817 (9)
H16B	0.135306	0.841437	−0.300355	0.218*	0.817 (9)
H16C	0.020894	0.736297	−0.253276	0.218*	0.817 (9)
C15A	0.100 (3)	0.759 (3)	−0.1873 (12)	0.136 (7)	0.183 (9)
H15C	0.029982	0.707524	−0.212092	0.163*	0.183 (9)
H15D	0.033784	0.825275	−0.161202	0.163*	0.183 (9)
C16A	0.217 (3)	0.825 (3)	−0.2484 (17)	0.154 (8)	0.183 (9)
H16D	0.282219	0.880321	−0.224439	0.230*	0.183 (9)
H16E	0.159884	0.876948	−0.287521	0.230*	0.183 (9)
H16F	0.286216	0.758960	−0.272898	0.230*	0.183 (9)
C17	0.22391 (17)	0.44660 (16)	0.57074 (9)	0.0470 (3)	
C18	0.05989 (19)	0.41770 (19)	0.61499 (11)	0.0601 (4)	
H18A	−0.013357	0.401095	0.577283	0.072*	
H18B	0.017668	0.495757	0.638985	0.072*	
C19	0.0595 (2)	0.30114 (19)	0.67900 (11)	0.0640 (4)	
H19A	−0.051773	0.281743	0.697837	0.077*	
H19B	0.109525	0.224332	0.656127	0.077*	
C20	0.1474 (3)	0.3242 (2)	0.74828 (12)	0.0728 (5)	
H20A	0.098793	0.402183	0.770390	0.087*	
H20B	0.259176	0.341852	0.729540	0.087*	
C21	0.1445 (3)	0.2098 (2)	0.81293 (13)	0.0855 (6)	
H21A	0.032790	0.187466	0.828121	0.103*	
H21B	0.200773	0.133945	0.791509	0.103*	
C22	0.2192 (4)	0.2331 (3)	0.88595 (14)	0.0931 (7)	
H22A	0.332013	0.252287	0.871187	0.112*	
H22B	0.165375	0.310539	0.906548	0.112*	
C23	0.2106 (4)	0.1204 (3)	0.95045 (15)	0.0981 (8)	
H23A	0.269654	0.044204	0.930842	0.118*	0.65 (5)
H23B	0.098298	0.097934	0.963297	0.118*	0.65 (5)
H23C	0.096991	0.104756	0.965914	0.118*	0.35 (5)
H23D	0.258004	0.042442	0.927930	0.118*	0.35 (5)
C24	0.277 (3)	0.1473 (18)	1.0246 (8)	0.099 (3)	0.65 (5)
H24A	0.387751	0.173991	1.010788	0.119*	0.65 (5)
H24B	0.215151	0.221683	1.044757	0.119*	0.65 (5)
C25	0.278 (3)	0.037 (2)	1.0898 (9)	0.121 (3)	0.65 (5)
H25A	0.167158	0.011833	1.104839	0.146*	0.65 (5)
H25B	0.337295	−0.038628	1.069280	0.146*	0.65 (5)
C26	0.348 (3)	0.062 (2)	1.1615 (8)	0.147 (4)	0.65 (5)
H26A	0.453304	0.096416	1.147258	0.221*	0.65 (5)
H26B	0.356735	−0.018961	1.195684	0.221*	0.65 (5)
H26C	0.278999	0.124964	1.188227	0.221*	0.65 (5)
C24A	0.288 (7)	0.130 (4)	1.0238 (16)	0.103 (5)	0.35 (5)
H24C	0.242291	0.207496	1.047626	0.124*	0.35 (5)
H24D	0.403103	0.142293	1.010066	0.124*	0.35 (5)
C25A	0.266 (6)	0.010 (3)	1.0832 (15)	0.109 (4)	0.35 (5)
H25C	0.151229	−0.004522	1.093009	0.131*	0.35 (5)
H25D	0.317531	−0.065179	1.059427	0.131*	0.35 (5)
C26A	0.329 (5)	0.013 (3)	1.1592 (14)	0.135 (6)	0.35 (5)
H26D	0.417268	−0.050794	1.164267	0.203*	0.35 (5)
H26E	0.244359	−0.007145	1.200983	0.203*	0.35 (5)
H26F	0.365797	0.099599	1.162455	0.203*	0.35 (5)

### Atomic displacement parameters (Å^2^)

**Table:**

	*U*^11^	*U*^22^	*U*^33^	*U*^12^	*U*^13^	*U*^23^
Cu1	0.04020 (10)	0.04349 (11)	0.04334 (11)	0.00564 (7)	−0.00618 (7)	−0.00991 (7)
O1	0.0724 (8)	0.0598 (7)	0.0527 (7)	0.0072 (6)	−0.0220 (6)	−0.0161 (5)
C1	0.0665 (10)	0.0556 (9)	0.0577 (10)	0.0108 (7)	−0.0171 (8)	−0.0146 (7)
N1	0.0456 (6)	0.0468 (6)	0.0507 (7)	0.0037 (5)	−0.0056 (5)	−0.0098 (5)
O2	0.0688 (7)	0.0586 (7)	0.0506 (6)	0.0051 (5)	−0.0158 (5)	−0.0155 (5)
C2	0.0698 (11)	0.0536 (9)	0.0720 (12)	0.0111 (8)	−0.0149 (9)	−0.0244 (8)
O3	0.0443 (6)	0.0550 (6)	0.0743 (8)	0.0055 (5)	0.0017 (5)	−0.0027 (6)
C3	0.0481 (8)	0.0403 (7)	0.0779 (11)	−0.0005 (6)	−0.0067 (7)	−0.0052 (7)
C4	0.0980 (15)	0.0636 (11)	0.0642 (12)	0.0209 (10)	−0.0256 (11)	−0.0040 (9)
O4	0.0457 (6)	0.0533 (6)	0.0640 (7)	0.0031 (4)	0.0037 (5)	−0.0065 (5)
C5	0.0950 (14)	0.0598 (10)	0.0554 (10)	0.0197 (9)	−0.0190 (9)	−0.0163 (8)
C7	0.0479 (8)	0.0607 (9)	0.0436 (8)	−0.0018 (6)	−0.0063 (6)	−0.0140 (7)
C6	0.0699 (12)	0.0428 (8)	0.1051 (16)	0.0052 (8)	−0.0181 (11)	−0.0060 (9)
C9	0.0872 (14)	0.0782 (12)	0.0510 (10)	−0.0010 (10)	−0.0159 (9)	−0.0133 (9)
C8	0.0976 (15)	0.0708 (12)	0.0519 (10)	−0.0043 (10)	−0.0215 (9)	−0.0163 (9)
C10	0.0892 (14)	0.0807 (13)	0.0520 (10)	−0.0051 (10)	−0.0187 (9)	−0.0104 (9)
C11	0.123 (2)	0.0867 (15)	0.0599 (12)	−0.0003 (14)	−0.0259 (12)	−0.0095 (11)
C12	0.1070 (18)	0.0885 (15)	0.0580 (11)	−0.0055 (13)	−0.0245 (11)	−0.0055 (10)
C13	0.147 (3)	0.0957 (17)	0.0661 (14)	0.0048 (17)	−0.0276 (15)	−0.0039 (12)
C14	0.137 (2)	0.114 (2)	0.0633 (14)	0.0056 (17)	−0.0300 (14)	0.0006 (13)
C15	0.152 (5)	0.116 (3)	0.077 (2)	−0.011 (3)	−0.033 (3)	0.010 (2)
C16	0.185 (5)	0.173 (5)	0.080 (3)	−0.016 (4)	−0.051 (3)	0.018 (3)
C15A	0.156 (14)	0.149 (13)	0.099 (11)	−0.016 (12)	−0.039 (11)	0.030 (10)
C16A	0.183 (17)	0.138 (16)	0.132 (16)	−0.014 (13)	−0.011 (14)	0.019 (13)
C17	0.0402 (7)	0.0567 (8)	0.0455 (8)	0.0005 (6)	−0.0056 (6)	−0.0112 (6)
C18	0.0415 (8)	0.0690 (10)	0.0682 (11)	0.0002 (7)	0.0024 (7)	−0.0100 (8)
C19	0.0535 (9)	0.0688 (11)	0.0685 (11)	−0.0131 (8)	0.0076 (8)	−0.0107 (9)
C20	0.0812 (13)	0.0715 (12)	0.0653 (12)	−0.0174 (10)	−0.0008 (10)	−0.0058 (9)
C21	0.1030 (17)	0.0797 (14)	0.0727 (14)	−0.0220 (12)	−0.0022 (12)	−0.0013 (11)
C22	0.1112 (19)	0.0910 (16)	0.0763 (15)	−0.0259 (14)	−0.0108 (13)	0.0051 (12)
C23	0.120 (2)	0.0962 (17)	0.0764 (15)	−0.0229 (15)	−0.0088 (14)	0.0046 (13)
C24	0.119 (5)	0.102 (6)	0.074 (4)	−0.015 (4)	−0.006 (4)	0.002 (3)
C25	0.157 (6)	0.112 (7)	0.093 (5)	−0.024 (5)	−0.016 (4)	0.006 (4)
C26	0.211 (9)	0.139 (11)	0.092 (4)	−0.031 (8)	−0.037 (5)	0.015 (5)
C24A	0.126 (9)	0.097 (8)	0.086 (8)	−0.021 (8)	−0.014 (7)	0.003 (6)
C25A	0.144 (8)	0.105 (9)	0.079 (6)	−0.023 (7)	−0.024 (6)	0.009 (6)
C26A	0.191 (14)	0.123 (14)	0.091 (7)	−0.015 (11)	−0.034 (7)	0.010 (8)

### Geometric parameters (Å, º)

**Table:**

Cu1—O4^i^	1.9708 (12)	C15—H15B	0.9700
Cu1—O2^i^	1.9725 (12)	C16—H16A	0.9600
Cu1—O3	1.9742 (13)	C16—H16B	0.9600
Cu1—O1	1.9785 (12)	C16—H16C	0.9600
Cu1—N1	2.1680 (14)	C15A—C16A	1.477 (17)
Cu1—Cu1^i^	2.6472 (8)	C15A—H15C	0.9700
O1—C7	1.253 (2)	C15A—H15D	0.9700
C1—N1	1.328 (2)	C16A—H16D	0.9600
C1—C2	1.375 (2)	C16A—H16E	0.9600
C1—H1	0.9300	C16A—H16F	0.9600
N1—C5	1.318 (2)	C17—C18	1.514 (2)
O2—C7	1.251 (2)	C18—C19	1.522 (3)
C2—C3	1.368 (3)	C18—H18A	0.9700
C2—H2	0.9300	C18—H18B	0.9700
O3—C17	1.245 (2)	C19—C20	1.508 (3)
C3—C4	1.374 (3)	C19—H19A	0.9700
C3—C6	1.498 (2)	C19—H19B	0.9700
C4—C5	1.380 (3)	C20—C21	1.513 (3)
C4—H4	0.9300	C20—H20A	0.9700
O4—C17	1.2586 (19)	C20—H20B	0.9700
C5—H5	0.9300	C21—C22	1.505 (3)
C7—C8	1.517 (2)	C21—H21A	0.9700
C6—H6A	0.9600	C21—H21B	0.9700
C6—H6B	0.9600	C22—C23	1.499 (3)
C6—H6C	0.9600	C22—H22A	0.9700
C9—C8	1.476 (3)	C22—H22B	0.9700
C9—C10	1.506 (3)	C23—C24A	1.491 (13)
C9—H9A	0.9700	C23—C24	1.501 (8)
C9—H9AB	0.9700	C23—H23A	0.9700
C8—H8A	0.9700	C23—H23B	0.9700
C8—H8AB	0.9700	C23—H23C	0.9700
C10—C11	1.485 (3)	C23—H23D	0.9700
C10—H10A	0.9700	C24—C25	1.494 (8)
C10—H10B	0.9700	C24—H24A	0.9700
C11—C12	1.491 (3)	C24—H24B	0.9700
C11—H11A	0.9700	C25—C26	1.469 (9)
C11—H11B	0.9700	C25—H25A	0.9700
C12—C13	1.479 (3)	C25—H25B	0.9700
C12—H12A	0.9700	C26—H26A	0.9600
C12—H12B	0.9700	C26—H26B	0.9600
C13—C14	1.470 (3)	C26—H26C	0.9600
C13—H13A	0.9700	C24A—C25A	1.505 (14)
C13—H13B	0.9700	C24A—H24C	0.9700
C14—C15A	1.509 (15)	C24A—H24D	0.9700
C14—C15	1.517 (5)	C25A—C26A	1.459 (13)
C14—H14A	0.9700	C25A—H25C	0.9700
C14—H14B	0.9700	C25A—H25D	0.9700
C14—H14C	0.9700	C26A—H26D	0.9600
C14—H14D	0.9700	C26A—H26E	0.9600
C15—C16	1.482 (6)	C26A—H26F	0.9600
C15—H15A	0.9700		
			
O4^i^—Cu1—O2^i^	90.45 (6)	C15—C16—H16A	109.5
O4^i^—Cu1—O3	167.70 (5)	C15—C16—H16B	109.5
O2^i^—Cu1—O3	88.57 (6)	H16A—C16—H16B	109.5
O4^i^—Cu1—O1	88.17 (6)	C15—C16—H16C	109.5
O2^i^—Cu1—O1	167.87 (5)	H16A—C16—H16C	109.5
O3—Cu1—O1	90.22 (6)	H16B—C16—H16C	109.5
O4^i^—Cu1—N1	99.38 (6)	C16A—C15A—C14	111.0 (18)
O2^i^—Cu1—N1	95.65 (6)	C16A—C15A—H15C	109.4
O3—Cu1—N1	92.92 (6)	C14—C15A—H15C	109.4
O1—Cu1—N1	96.47 (6)	C16A—C15A—H15D	109.4
O4^i^—Cu1—Cu1^i^	84.31 (4)	C14—C15A—H15D	109.4
O2^i^—Cu1—Cu1^i^	83.26 (5)	H15C—C15A—H15D	108.0
O3—Cu1—Cu1^i^	83.39 (4)	C15A—C16A—H16D	109.5
O1—Cu1—Cu1^i^	84.61 (5)	C15A—C16A—H16E	109.5
N1—Cu1—Cu1^i^	176.17 (4)	H16D—C16A—H16E	109.5
C7—O1—Cu1	122.09 (11)	C15A—C16A—H16F	109.5
N1—C1—C2	123.45 (17)	H16D—C16A—H16F	109.5
N1—C1—H1	118.3	H16E—C16A—H16F	109.5
C2—C1—H1	118.3	O3—C17—O4	125.37 (14)
C5—N1—C1	116.58 (15)	O3—C17—C18	116.84 (14)
C5—N1—Cu1	121.66 (12)	O4—C17—C18	117.78 (14)
C1—N1—Cu1	121.03 (11)	C17—C18—C19	115.08 (14)
C7—O2—Cu1^i^	124.04 (11)	C17—C18—H18A	108.5
C3—C2—C1	120.18 (17)	C19—C18—H18A	108.5
C3—C2—H2	119.9	C17—C18—H18B	108.5
C1—C2—H2	119.9	C19—C18—H18B	108.5
C17—O3—Cu1	124.01 (10)	H18A—C18—H18B	107.5
C2—C3—C4	116.31 (16)	C20—C19—C18	113.70 (16)
C2—C3—C6	121.37 (17)	C20—C19—H19A	108.8
C4—C3—C6	122.31 (18)	C18—C19—H19A	108.8
C3—C4—C5	120.28 (18)	C20—C19—H19B	108.8
C3—C4—H4	119.9	C18—C19—H19B	108.8
C5—C4—H4	119.9	H19A—C19—H19B	107.7
C17—O4—Cu1^i^	122.79 (11)	C19—C20—C21	113.99 (17)
N1—C5—C4	123.21 (18)	C19—C20—H20A	108.8
N1—C5—H5	118.4	C21—C20—H20A	108.8
C4—C5—H5	118.4	C19—C20—H20B	108.8
O2—C7—O1	125.93 (14)	C21—C20—H20B	108.8
O2—C7—C8	116.68 (15)	H20A—C20—H20B	107.6
O1—C7—C8	117.36 (15)	C22—C21—C20	115.62 (19)
C3—C6—H6A	109.5	C22—C21—H21A	108.4
C3—C6—H6B	109.5	C20—C21—H21A	108.4
H6A—C6—H6B	109.5	C22—C21—H21B	108.4
C3—C6—H6C	109.5	C20—C21—H21B	108.4
H6A—C6—H6C	109.5	H21A—C21—H21B	107.4
H6B—C6—H6C	109.5	C23—C22—C21	115.0 (2)
C8—C9—C10	115.20 (18)	C23—C22—H22A	108.5
C8—C9—H9A	108.5	C21—C22—H22A	108.5
C10—C9—H9A	108.5	C23—C22—H22B	108.5
C8—C9—H9AB	108.5	C21—C22—H22B	108.5
C10—C9—H9AB	108.5	H22A—C22—H22B	107.5
H9A—C9—H9AB	107.5	C24A—C23—C22	119.4 (11)
C9—C8—C7	116.89 (16)	C22—C23—C24	114.5 (6)
C9—C8—H8A	108.1	C22—C23—H23A	108.6
C7—C8—H8A	108.1	C24—C23—H23A	108.6
C9—C8—H8AB	108.1	C22—C23—H23B	108.6
C7—C8—H8AB	108.1	C24—C23—H23B	108.6
H8A—C8—H8AB	107.3	H23A—C23—H23B	107.6
C11—C10—C9	115.87 (19)	C24A—C23—H23C	107.5
C11—C10—H10A	108.3	C22—C23—H23C	107.5
C9—C10—H10A	108.3	C24A—C23—H23D	107.5
C11—C10—H10B	108.3	C22—C23—H23D	107.5
C9—C10—H10B	108.3	H23C—C23—H23D	107.0
H10A—C10—H10B	107.4	C25—C24—C23	117.0 (10)
C10—C11—C12	117.3 (2)	C25—C24—H24A	108.1
C10—C11—H11A	108.0	C23—C24—H24A	108.1
C12—C11—H11A	108.0	C25—C24—H24B	108.1
C10—C11—H11B	108.0	C23—C24—H24B	108.1
C12—C11—H11B	108.0	H24A—C24—H24B	107.3
H11A—C11—H11B	107.2	C26—C25—C24	116.9 (9)
C13—C12—C11	116.2 (2)	C26—C25—H25A	108.1
C13—C12—H12A	108.2	C24—C25—H25A	108.1
C11—C12—H12A	108.2	C26—C25—H25B	108.1
C13—C12—H12B	108.2	C24—C25—H25B	108.1
C11—C12—H12B	108.2	H25A—C25—H25B	107.3
H12A—C12—H12B	107.4	C25—C26—H26A	109.5
C14—C13—C12	117.9 (3)	C25—C26—H26B	109.5
C14—C13—H13A	107.8	H26A—C26—H26B	109.5
C12—C13—H13A	107.8	C25—C26—H26C	109.5
C14—C13—H13B	107.8	H26A—C26—H26C	109.5
C12—C13—H13B	107.8	H26B—C26—H26C	109.5
H13A—C13—H13B	107.2	C23—C24A—C25A	112.8 (17)
C13—C14—C15A	131.2 (13)	C23—C24A—H24C	109.0
C13—C14—C15	114.4 (3)	C25A—C24A—H24C	109.0
C13—C14—H14A	108.7	C23—C24A—H24D	109.0
C15—C14—H14A	108.7	C25A—C24A—H24D	109.0
C13—C14—H14B	108.7	H24C—C24A—H24D	107.8
C15—C14—H14B	108.7	C26A—C25A—C24A	117.3 (17)
H14A—C14—H14B	107.6	C26A—C25A—H25C	108.0
C13—C14—H14C	104.4	C24A—C25A—H25C	108.0
C15A—C14—H14C	104.4	C26A—C25A—H25D	108.0
C13—C14—H14D	104.4	C24A—C25A—H25D	108.0
C15A—C14—H14D	104.4	H25C—C25A—H25D	107.2
H14C—C14—H14D	105.6	C25A—C26A—H26D	109.5
C16—C15—C14	113.3 (5)	C25A—C26A—H26E	109.5
C16—C15—H15A	108.9	H26D—C26A—H26E	109.5
C14—C15—H15A	108.9	C25A—C26A—H26F	109.5
C16—C15—H15B	108.9	H26D—C26A—H26F	109.5
C14—C15—H15B	108.9	H26E—C26A—H26F	109.5
H15A—C15—H15B	107.7		

Symmetry code: (i) −*x*+1, −*y*+1, −*z*+1.

### Hydrogen-bond geometry (Å, º)

*Cg* is the centroid of the N1/C1–C5 ring.

**Table:**

*D*—H···*A*	*D*—H	H···*A*	*D*···*A*	*D*—H···*A*
C6—H6*B*···O2^ii^	0.96	2.73	3.577	148
C18—H18*A*···O3^iii^	0.97	2.90	3.841	163
C6—H6*C*···*Cg*1^iv^	0.96	2.94	3.665 (2)	134

Symmetry codes: (ii) *x*, *y*−1, *z*; (iii) −*x*, −*y*+1, −*z*+1; (iv) −*x*, −*y*+2, −*z*+1.
